# Courtship Behavior and Induced Spawning Dynamics of the Freshwater Shark, *Wallago attu*, in Captivity: Implications for Silurid Reproductive Strategies

**DOI:** 10.3390/ani16203296

**Published:** 2026-10-20

**Authors:** Saeed Akram Khan, Muhammad Khalil Ahmad Khan, Shahid Sherzada, Muhammad Mahd Nazir, Rabia Yaseen, Muhammad Bilal Bhatti, Muhammad Shahid Majeed, Sania Riaz, Abdallah Tageldein Mansour, Talib Hussain, Joanna Nowosad, Dariusz Kucharczyk

**Affiliations:** 1 Department of Zoology, Government College University, Lahore 54000, Pakistan; saeed.akram@gcu.edu.pk (S.A.K.); shahid.sherzada@gcu.edu.pk (S.S.); muhammadmahdnazir@gmail.com (M.M.N.); cbiach1234@gmail.com (R.Y.); shahidmajeed1767254@gmail.com (M.S.M.); saniariaz919@gmail.com (S.R.); 2 Department of Zoology, University of Okara, Okara 56300, Pakistan; dr.khalil@uo.edu.pk; 3 Higher Institution Centre of Excellent, Institute of Tropical Aquaculture and Fisheries, Universiti Malaysia Terengganu, Terengganu, Kuala Nerus 21030, Malaysia; p6292@pps.umt.edu.my; 4 Animal and Fish Production Department, College of Agricultural and Food Sciences, King Faisal University, Al-Ahsa 31982, Saudi Arabia; 5 Department of Aquatic Resources Management, Faculty of Fisheries and Marine Science, Universitas Diponegoro, Semarang 50275, Indonesia; talibhussain@students.undip.ac.id; 6 Department of Research and Development, ChemProf, Gutkowo 54B, 11-041 Olsztyn, Poland; j.nowosad@infish.com.pl; 7 Department of Ichthyology, Hydrobiology and Aquatic Ecology, National Inland Fisheries Research Insttute, Al. Oczapowskiego 10, 10-719 Olsztyn, Poland

**Keywords:** *Wallago attu*, courtship behavior, male dominance, U-shaped wrapping, captive breeding

## Abstract

The vulnerable catfish *Wallago attu*, a commercially important freshwater species in Southeast Asia, faces population decline due to habitat degradation and overfishing. This study is the first to document the courtship behavior and induced spawning of *W. attu* under captive conditions in circular spawning tanks. The hormonal induction was achieved using a combination of salmon gonadotropin-releasing hormone analogue and domperidone (0.5 mL kg^−1^ for females; 0.2 mL kg^−1^ for males). Males exhibited dominant courtship behavior, characterized by aggressive chasing and U-shaped body wrapping around the female’s abdomen for 5.15 ± 0.17 s, leading to synchronized gamete release. The latency period prior to the onset of spawning occurred within a time frame of 9–10 h following hormonal administration. The findings indicate that the removal of brooders following egg release resulted in a significantly higher rate of fertilization and hatching, with a percentage of 88.12 ± 0.26%, as compared to 67.78 ± 1.78% in tanks that contained brooders This observation suggests the presence of egg predation. Plastic strings functioned as effective artificial substrates, enhancing fertilization (86.42 ± 1.18%) and hatching (89.28 ± 0.80%) in comparison to aquatic weeds. The findings presented herein provide a foundation of information upon which the establishment of efficient captive breeding and hatchery protocols for *W. attu* can be based. Such protocols will in turn support the conservation of the species and the sustainable development of aquaculture.

## Simple Summary

This study constitutes the inaugural global documentation of the courtship behavior exhibited by the vulnerable catfish, *Wallago attu*, within the context of captivity. A robust male-driven mating system was identified, characterized by the aggressive courtship behavior of males, which entailed wrapping their bodies in a distinctive U-shape around the female abdomen. This behavior was observed to serve the purpose of coordinating the synchronized release of eggs and sperm. The removal of adult fish from the spawning tanks post-egg-laying resulted in a significant enhancement in the hatching rate. This finding suggests the possibility of brooders consuming their own eggs. Furthermore, the utilization of simple plastic strings as spawning substrates for egg attachment has been demonstrated to be more efficacious than natural aquatic weeds. Within a few hours of the injection, the entire courtship-to-spawning cycle was completed. The practical, cost-effective results offer hatchery managers and conservationists a viable approach to the breeding of this vulnerable species, thereby reducing the pressure on wild populations and encouraging sustainable aquaculture.

## 1. Introduction

Captive breeding of catfish necessitates hormonal and thermal stimulation [1,2,3]. In the case of females, the eggs are obtained by hand-stripping. However, for males, the situation is much more complicated, as it is impossible to obtain sufficient sperm through abdominal massage or, for example, the use of catheters [1,4,5]. The male subjects are usually euthanized, after which the testicles are harvested. Subsequent to the process of maceration, the milt is extracted and then amalgamated with remnants of testicular tissue. This mixture is utilized either in a single application or in batches for the purpose of fertilizing eggs [1,3,6]. From the perspectives of both aquaculture and the genetic variability of offspring, two disadvantages are associated with artificial reproduction. Firstly, there is an irreversible loss of males. Secondly, the process entails the utilization of sperm from a single male individual for the purpose of fertilizing ova from multiple female subjects. Consequently, particularly in the context of introducing novel species of catfish into aquaculture, where the number of spawners (including males) is often limited, alternative methods of breeding these fish in captivity are investigated to minimize the removal of males from breeding stocks [4,5,6].

*Wallago attu* (locally referred to as “Malli”) is a species of commercial importance, with a price of approximately 1300 Pakistani rupees per kilogram [kg] (4.7 USD per kg) in local markets. This species is considered to be of significant commercial importance in Asia, where it is found in riverine waters [7]. The Asian silurid catfish (*Wallago attu*) is widely distributed throughout the Indian subcontinent and several Southeast Asian countries, including Thailand, Laos, Cambodia, Vietnam, Malaysia, and Indonesia [8]. *W. attu*: The population in this region is in decline. The species is listed as Vulnerable (IUCN Red List) due to the following factors: habitat degradation, overfishing, and pollution. Recent genetic research has confirmed the presence of bottlenecks and reduced population sizes in Pakistani rivers [7,9].

In natural habitats, animals utilize sensory information to attract mates of the opposite sex. The term “courtship” is used to denote the aggregate of these behavioral interactions. The term “courtship behavior” is employed to denote all types of interactions between males and females that contribute to the process of spawning [10]. In the animal kingdom, species employ a variety of signals to attract mates. This behavior, which has been termed “courtship behavior”, is a fundamental aspect of reproductive strategy. In fish species, a specific sequence of interactions between males and females is observed to facilitate successful spawning. Such interactions include chasing, body movements, and changes in coloration [11,12,13]. The numerous and varied courtship patterns exhibited by different species encompass not only straightforward pair swimming but also more intricate behavioral displays. These patterns are contingent on factors such as body size, environmental conditions, reproductive status, and prior breeding experience [14,15,16]. It is evident that these behavioral interactions are of paramount importance in ensuring the synchronization of gamete release and reproductive success. It is well established that courtship behavior is of crucial importance in the context of fish reproduction. However, there is a paucity of data available on the behavioral dynamics of *Wallago attu*, particularly in the context of captive settings.

The degree of reproductive success in fish is contingent on behavioral synchronization between males and females, encompassing species-specific courtship and spawning interactions. The complex signaling, chasing, and body movements involved in courtship behavior are designed to facilitate gamete release and fertilization. These behaviors are frequently modulated by hormonal and environmental cues [17]. It is imperative to comprehend such behavioral patterns to optimize induced breeding protocols in catfishes and to ensure high fertilization and hatching success under captive conditions [18]. This report constitutes the inaugural documentation of the courtship and mating behavior exhibited by *W. attu* in captivity within the geographical confines of Pakistan. It is noteworthy that no extant documentation exists pertaining to the behavioral and reproductive dynamics of this species in captivity. In view of the fact that the species is in a vulnerable state and its wild populations are in decline, it is imperative to gain a more profound understanding of its reproductive behavior. The objectives of the present study were threefold: first, to observe courtship behavior; second, to document mating rituals; and third, to evaluate egg hatchability with and without parental presence. The findings of this study provide valuable insights into the reproductive biology of *W. attu*.

## 2. Materials and Methods

### 2.1. Brooder Selection and Acclimatization

The *W. attu* specimens were obtained from the Balloki headworks in the Punjab region of Pakistan. Adult individuals (*n* = 8, 4 pairs) were collected from natural water bodies in the month of March and maintained in ponds for a period of 3 months. The fish were collected as by-catch from local fishermen operating in the vicinity of the Balloki headworks in Punjab, Pakistan. These fish were maintained in earthen ponds for a period of three months (temperature: 24–28 °C; pH 7.4–7.9). During this period, the fish were provided with sustenance on two occasions each day (09:00 am and 05:00 pm) at a rate equivalent to 3% of their body weight, in the form of a commercial diet (50% CP) (see Table 1 for details). The fish were subsequently transferred from ponds and maintained in rectangular tanks under hatchery conditions at the Sindhuan Fish Seed Hatchery in Head Balloki, Punjab, Pakistan. The fish were maintained at a 1:1 male to female ratio.

Sexual dimorphism was evident in both male and female fish. It was observed that the male subjects displayed a pointed red genital papilla. Adult females displayed rounded papillae in close proximity to the vent. It has been observed that, during the designated breeding season, the abdominal region of the female subject is extended from the body axis. The presence of rough pectoral fins was a distinguishing feature of the male specimens [19].

### 2.2. Induced Spawning Setup

The details of the research setup are presented in Table 2. Prior to hormonal administration, the subjects’ weight, length, and age were meticulously calculated (see Table 3, Table 4, Table 5, Table 6, Table 7 and Table 8).

#### 2.2.1. Hormonal Induction

Each brooder was meticulously netted, anesthetized with clove oil (30 mg/L), and weighed prior to being injected intramuscularly in the dorsal musculature with Ovaprim. The fish that had been administered the injection were then returned to the recovery tanks, where they were observed until they were able to swim in a normal manner. Each injection was completed within five minutes in order to minimize stress.

The fish were injected with Ovaprim (sGnRHa + domperidone) at a dose of 0.5 mL kg^−1^ live body weight for females and 0.2 mL kg^−1^ live body weight for males [20,21,22]. The dosage of the medication was calculated based on the patient’s body weight, as outlined in Table 3. The fish were subsequently placed in circular tanks, with each tank housing a pair of fish, in order to observe any potential courtship and mating rituals.

#### 2.2.2. Physicochemical Parameters

The physicochemical parameters were recorded during the whole trial, and statistical data are presented in Table 9.

### 2.3. Courtship Observation Strategy

The observations were conducted at random intervals, with 1–2 h of video recordings obtained during each observation period. The major stages of courtship behavior were subsequently identified, measured, and described. A total of four pairs of fish were observed during the courtship behavior.

### 2.4. Time-Lapse Imaging

The courtship and mating behavior of the subjects was assessed. The administration of hormonal doses was followed by the observation of the fish at several intervals. The courtship and mating processes were documented using video recording equipment to produce time-lapse sequences that captured the entirety of the reproductive process. The duration of each courtship behavior was calculated separately.

### 2.5. Effect of Brooder Presence on Egg Survival

The impact of brooder presence on egg survival was evaluated through two experimental approaches. In the first approach, male and female brooders were retained within a circular spawning tank following egg release. In the second approach, the brooders were immediately removed post-spawning. In both cases, the fertilization and hatching rates were calculated in order to assess whether the presence of the brooder had an effect on the survival rate of the eggs.

The presence of fish brooders inside the fish tank after egg release is indicated by T1.

T2: The process of removing the brooders from the fish tank following the release of the eggs.

### 2.6. Spawning Substrates and Preparation

The adhesive eggs were provided with attachment substrates of natural or artificial origin. The natural substrate consisted of *Hydrilla verticillata* collected in the fresh state, which was thoroughly rinsed and surface disinfected in a potassium permanganate solution (10 mg/L for 30–60 min), followed by repeated washing before placement in spawning tanks. The artificial substrate comprised white nylon plastic strings, arranged as bundled strands and fixed to a frame. These were suspended in the tank to create a dense attachment surface.

The present study set out to evaluate the effectiveness of plastic strings as an alternative spawning substrate to aquatic weeds. In order to achieve this, the experimenter provided either plastic strings or aquatic weeds in separate tanks and then compared fertilization and hatching rates between the treatments.

The first hypothesis (P0) states that aquatic weeds act as a substrate for adhesive eggs. The second hypothesis (P1) states that plastic strings act as a substrate for adhesive eggs.

### 2.7. Determination of Fertilization and Hatching Rate

In order to ascertain the fertilization rate, egg samples were collected on six occasions from discrete locations within each tank. The samples were then transferred to 500 mL beakers for examination. It should be noted that the samples were collected from different locations within the tank. The collected samples were then subjected to examination under a light source. The observation of fertilized eggs was conducted under the microscope in accordance with the established procedure [23].

The fertilization rate was measured by the following formula:

Fertilization rate (%) = (Number of fertilized eggs in sample/Total number of eggs) × 100

The hatching rate was calculated using the formula in [24].

Hatching rate (%) = (Number of hatching eggs in sample/Total number of fertilized eggs) × 100

### 2.8. Reproductive Parameters

#### 2.8.1. Spawning Pattern

The observed reproductive activity over the spawning period was then assessed in order to determine whether spawning occurred in one distinct event or multiple batches.

#### 2.8.2. Spawning Frequency

Monitored spawning events over the course of the entire reproductive cycle.

#### 2.8.3. Spawning Period

The following data were recorded: dates of spawning and egg release. The following summary is based on observations of the beginning, peak, and end.

#### 2.8.4. Spawning Associated Temperature

The temperature of the water in the study tanks was measured using a calibrated thermometer.

### 2.9. Statistical Analysis

The data pertaining to fish weight and hormonal dose were analyzed using a factorial ANOVA. The model incorporated treatment (T1 vs. T2) or (P0 vs. P1) and sex (male vs. female) as the primary factors, with their interaction incorporated into the analysis. Nevertheless, the analysis of the data pertaining to physicochemical parameters and reproductive performance was conducted by employing the one-way ANOVA technique. The General Linear Model procedure was implemented in the SAS software (version 9.1). In order to facilitate the comparison of significant treatment means, the Duncan multiple range test was applied, with a significance level of *p* ≤ 0.05.

## 3. Results

### 3.1. Spawning and Mating Behavior in Wallago attu

#### 3.1.1. Courtship Observations

**Figure 1:**
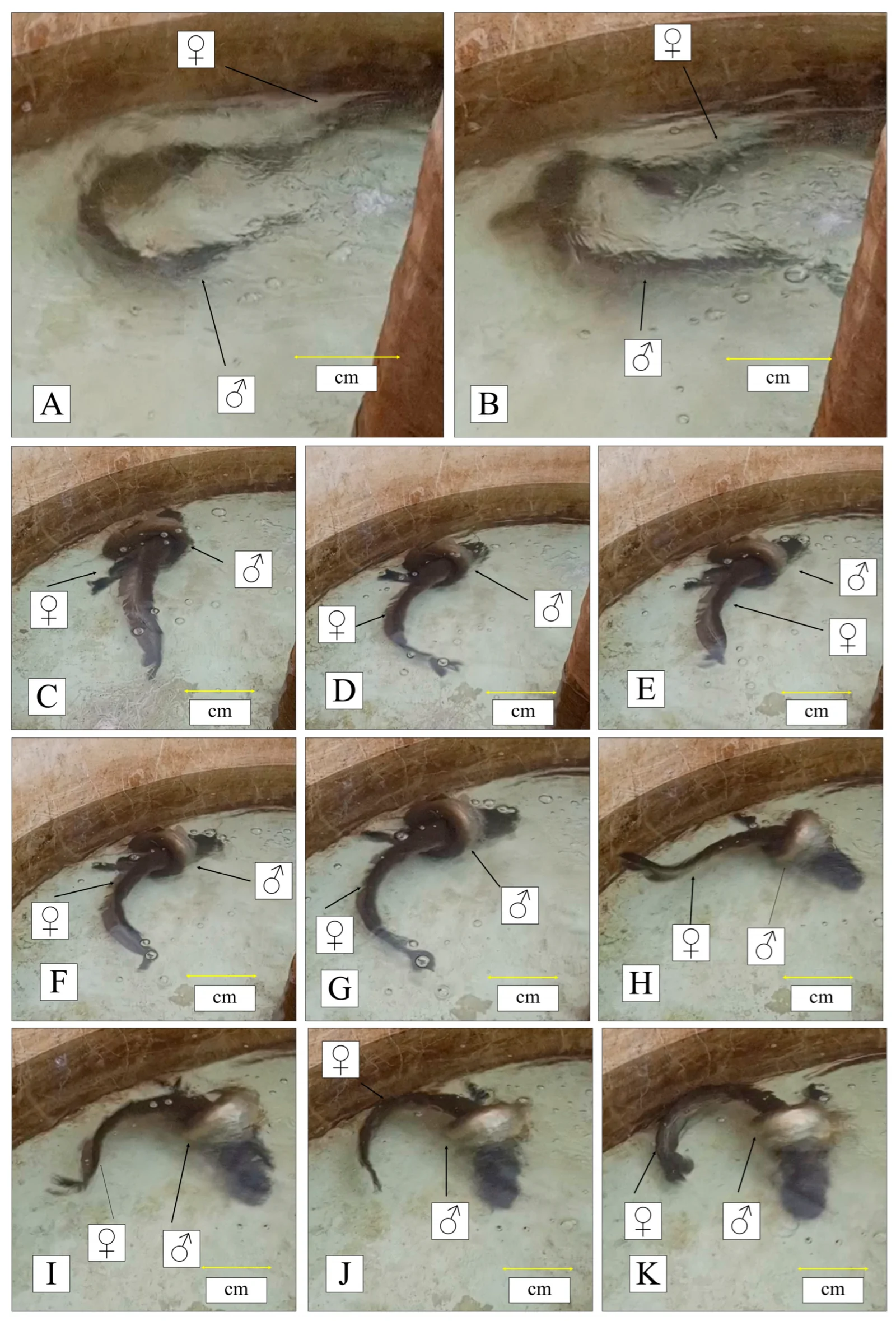
Male fish chasing female fish of *W. attu* during induced breeding (**A**,**B**); U-shaped bending of male fish around female fish (**C**–**K**). The yellow arrow indicates the scale bar (1 cm = 30 cm).

**Figure 2:**
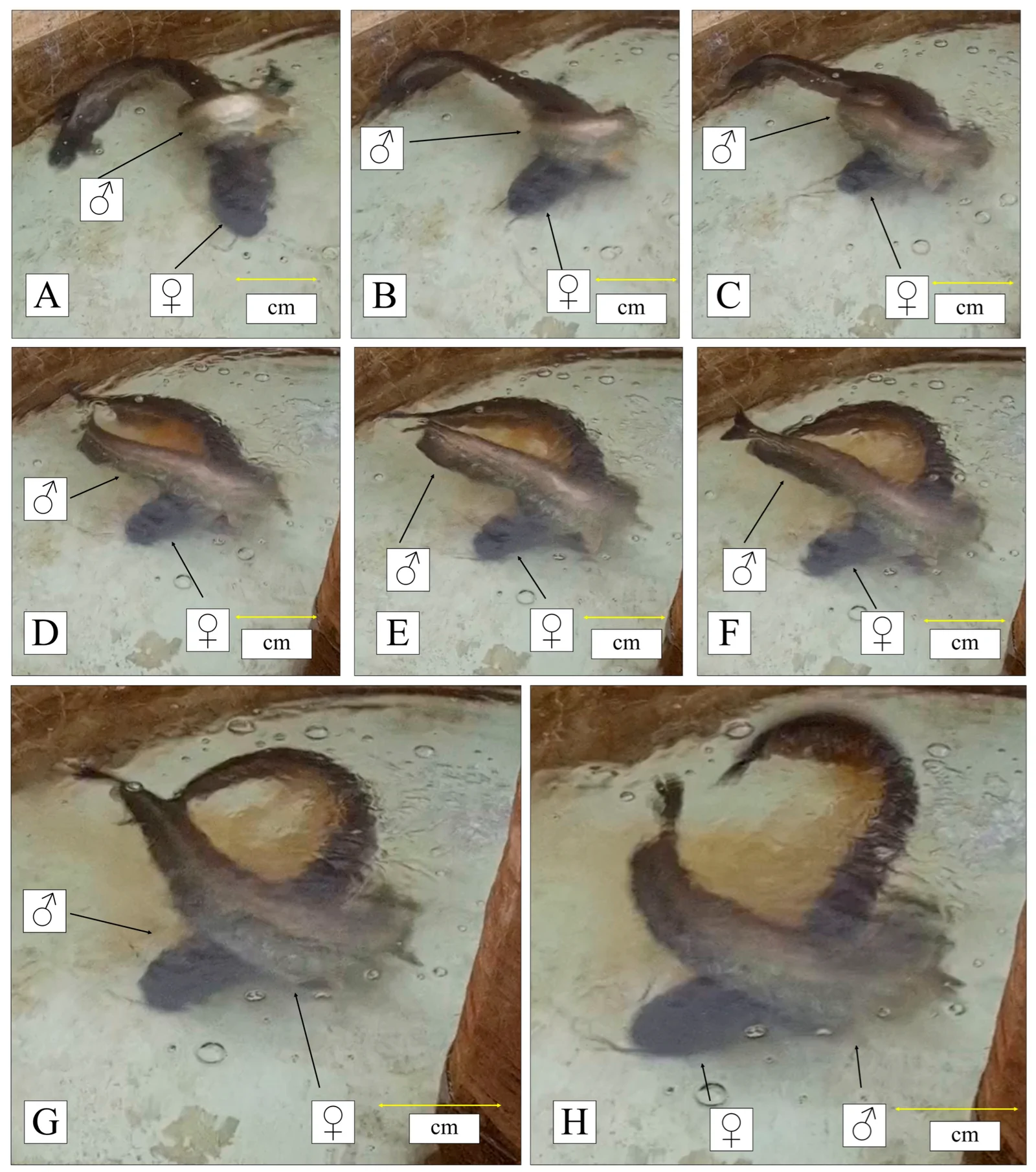
Bending bodies of female fish of *W. attu* during induced breeding (**A**–**C**); female fish releasing eggs (**D**–**H**). The yellow arrow indicates the scale bar (1 cm = 30 cm).

**Figure 3:**
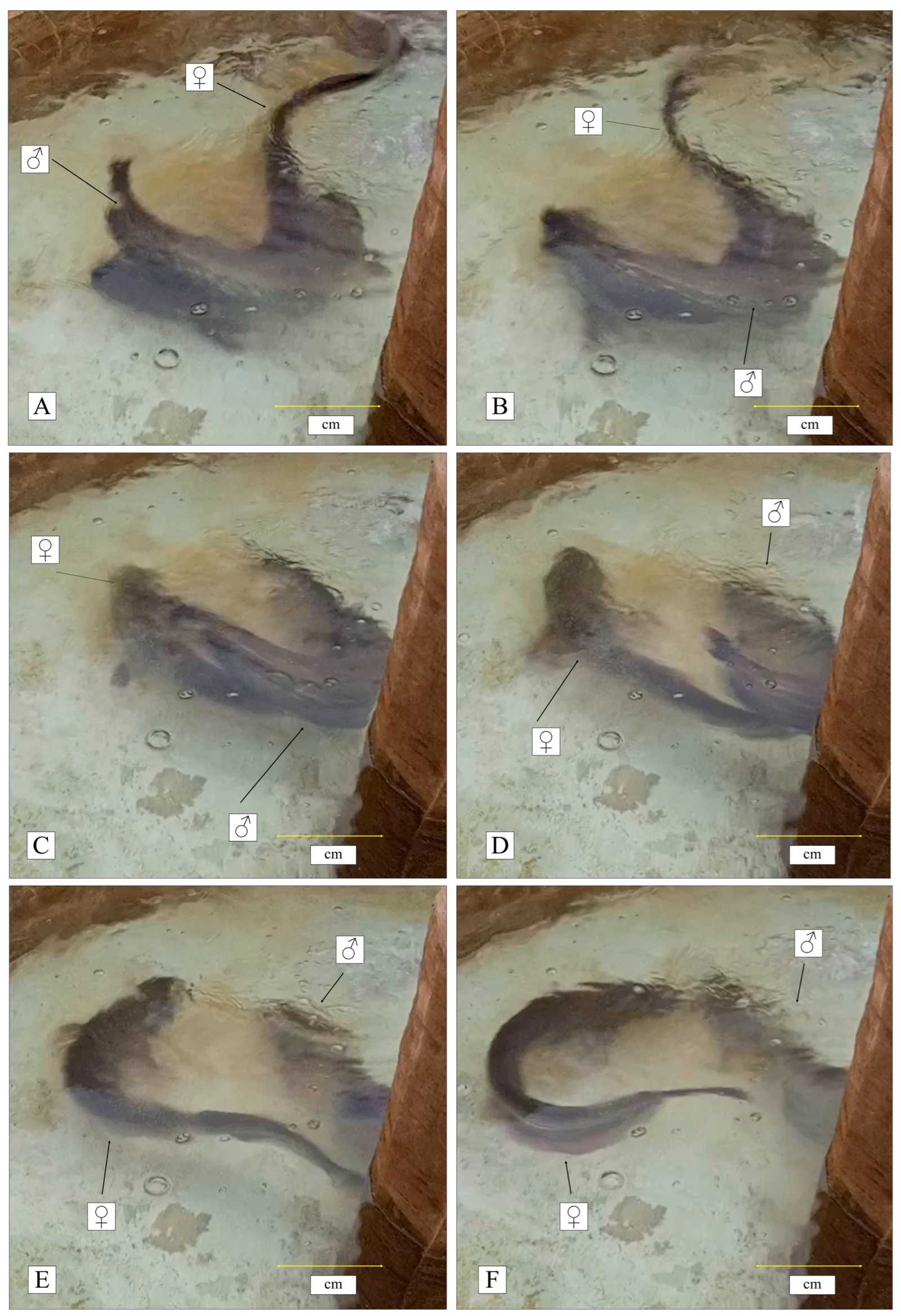
Male and female fish of *Wallago attu* during induced breeding, separated after egg release (**A**–**F**). The yellow arrow indicates the scale bar (1 cm = 30 cm).

**Figure 4:**
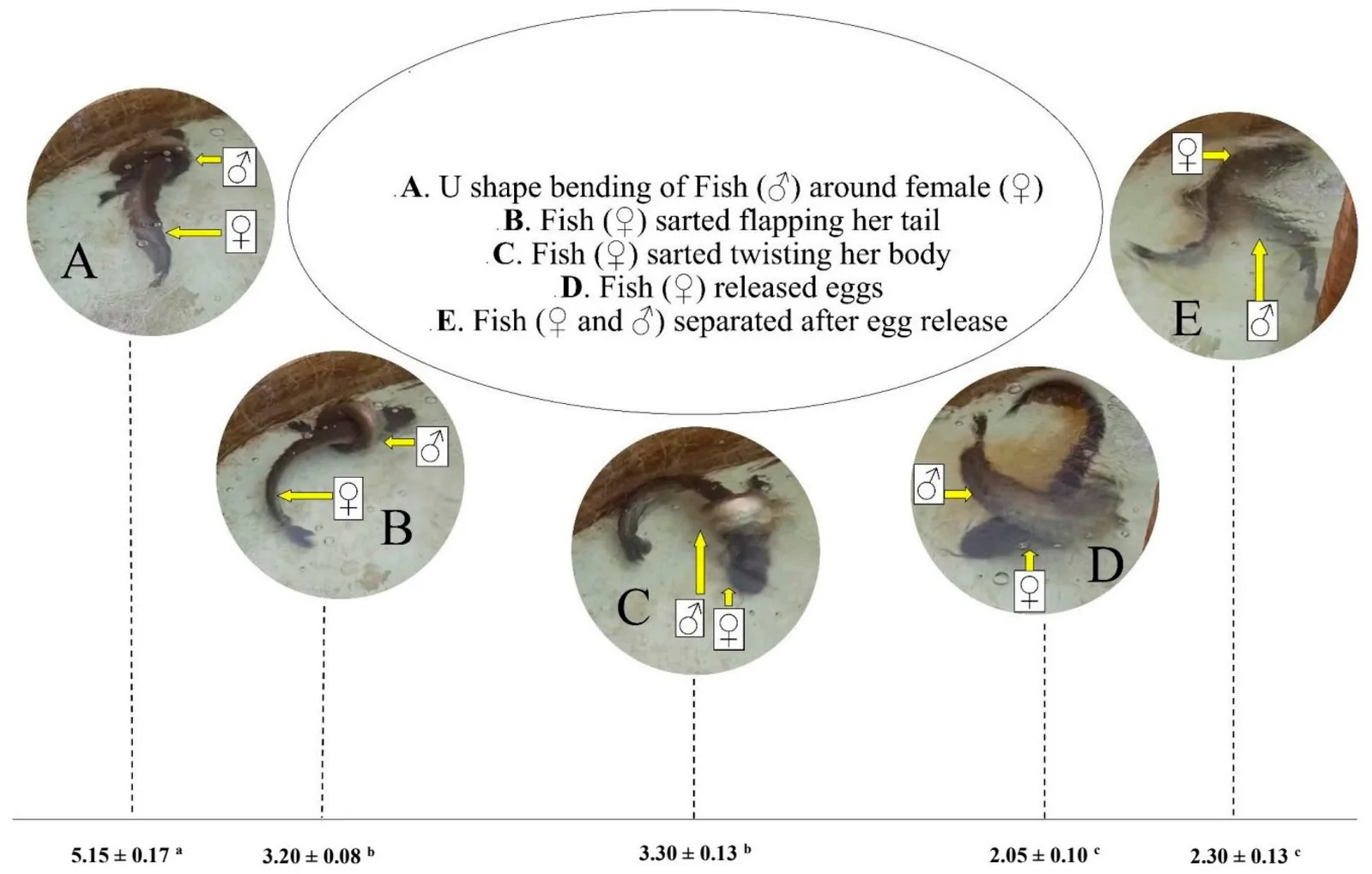
Occurrence of courtship events during trials (mean duration (seconds) ± SD) of behavioral events of *W. attu* during induced breeding: (**A**) U-shaped bending of male fish around female fish; (**B**) female fish started flapping her tail; (**C**) female fish started twisting her body; (**D**) female fish released eggs; (**E**) male and female fish separated after egg release. Superscripts show the significant difference among treatments (courtship events).

**Figure 5:**
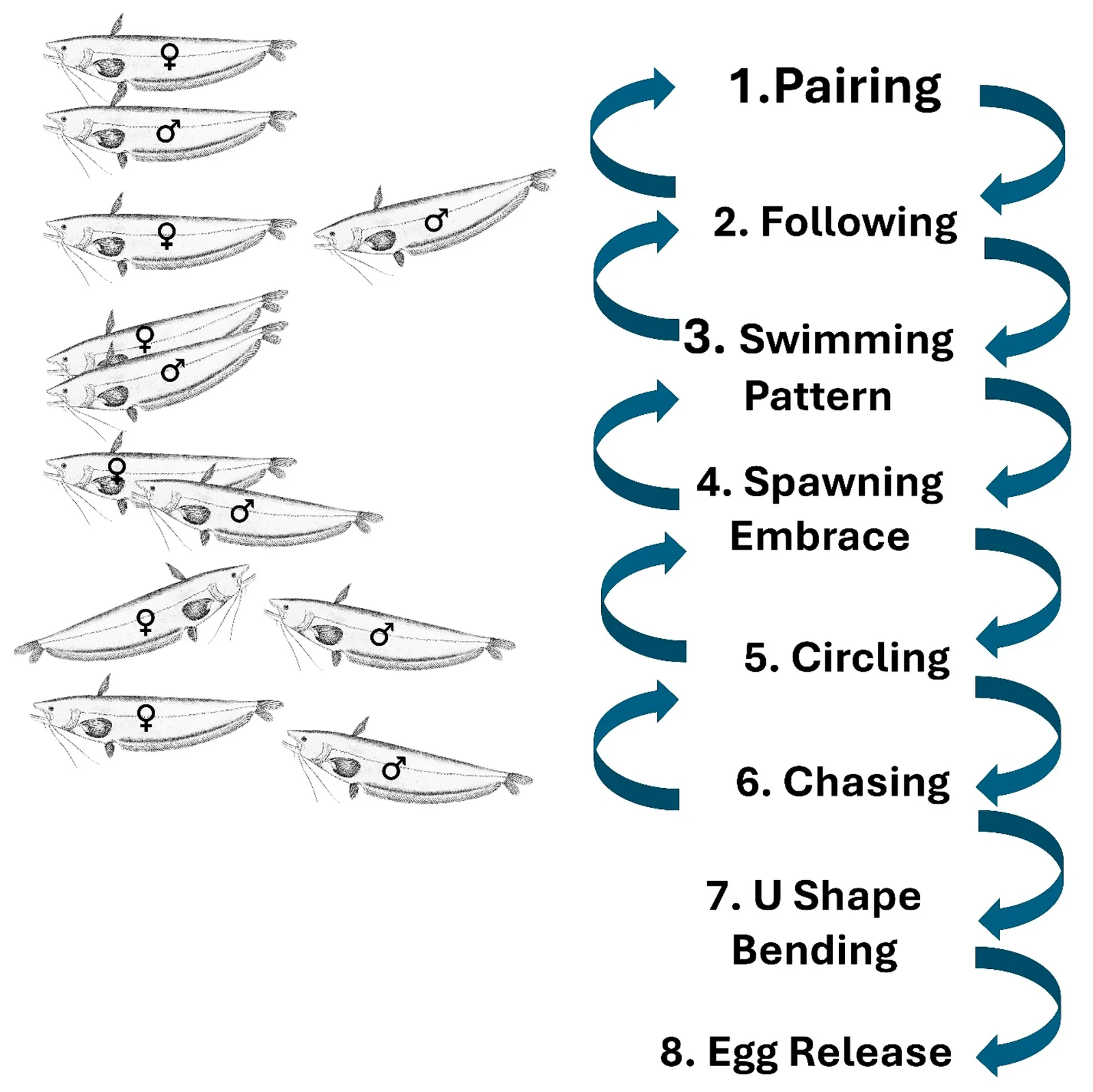
Sequence of fish positions before and during courtship and mating of *Wallago attu* during induced breeding: 1. Before the first courtship attempts, males and females were lying motionless and separated on the bottom of the tank. The female is easily identified by her large, bloated stomach. 2. Male fish followed female fish in the circular tank. 3. The swimming pattern included side-to-side swimming and often in a circular motion. 4. The male fish was observed rubbing his body against the female fish’s abdomen. 5. Pairs were observed swimming in circles. 6. The male chased aggressively and forced the female fish to copulate. 7. U-shaped bending of the male fish around the female fish’s belly. 8. The female fish released eggs in the tank. Sequences of fish positions are mentioned in Figure 5, showing illustrations of various events before and during courtship activity.

It was observed that *W. attu* brooders began to demonstrate courtship behavior following the administration of a hormonal dose over a period of 3.5 h. Initially, the male fish remained at the base of the circular tank in proximity to the female fish. As time progressed, a marked tendency for movement was observed among the fish, both male and female, within the circular tank. These movements resulted in chasing activity, in which male fish pursued female fish (see Figure 1A,B). It has been demonstrated that the male specimen displaying greater levels of activity and aggression is the one that is paired with the female in the breeding tank. The male was observed to strike the female’s snout and vent region with greater frequency. It is important to note that mating was preceded by elaborate courtship. The male subject was observed to pursue the female, and the paired brooders exhibited a marked increase in activity between 4.5 and 6 h following the administration of the hormone injection (see Figure 1A,B). The process of spawning continued until the release of gametes. During the period of courtship, the male would adopt a curved posture around the female, thus bringing the pair into close proximity. Subsequently, the process of spawning occurred, with the male releasing milt while the female released eggs, resulting in external fertilization (see Figure 1C–K and Figure 2A–H and Table 10). The eggs were deposited in a transparent area and were held by disinfected nylon threads. Following the release of the eggs, a separation of the male and female fish was observed (see Figure 3). The courtship behavior timings (seconds) are displayed in Figure 4. The various courtship behaviour patterns are depicted in Figure 5.

Prior to the act of spawning, the male exhibited aggressive behavior, which included chasing the female and ultimately enveloping her in a U-shaped posture (see Table 11 for further details).

#### 3.1.2. Egg Survival in Relation to Brooder Presence

In this study, we sought to compare the outcomes of fertilization and hatching, contingent on the retention (T1) or removal (T2) of brooders post-spawning. The level of fertilization was found to be higher in cases where brooders were retained in the tank (82.25 ± 2.44%) in comparison to cases where they were removed (70.77 ± 2.50%). The data reveal a higher hatching success when brooders were removed (T2, 88.12 ± 0.26%) in comparison to the retention group (T1, 67.78 ± 1.78%), as illustrated in Table 12.

### 3.2. Reproductive Performance on Different Spawning Surfaces

A further comparison was made between the success of spawning on plastic strings versus aquatic weeds. Tanks equipped with plastic strings (P1) exhibited higher fertilization and hatching rates of 86.42 ± 1.18% and 89.28 ± 0.80%, respectively. In contrast, tanks infested with aquatic weeds (P0) exhibited reduced rates of weed proliferation. The mean fertilization rate was 75.16 ± 2.26%, and the mean hatching rate was 69.53 ± 3.13%, as shown in Table 13. The findings indicate that the utilization of plastic strings resulted in the provision of a clean and stable surface for egg attachment, which may have contributed to a reduction in egg loss.

## 4. Discussion

There are no records of courtship and mating rituals in *W. attu*. The literature only includes induced spawning and early development; no detailed behavioral accounts exist [17,22]. Thus, the present study provides important insights into the reproductive behavior of *W. attu* and contributes to a broader understanding of reproductive strategies in Siluridae. A distinct male-dominated courtship sequence was observed. This sequence was characterized by physical alignment, persistent chasing, and a brief U-shaped clasp immediately before gamete release. These features reflect the species-specific and high-energy mating tactics within externally fertilizing catfishes.

### 4.1. Male Dominance and Courtship Mechanics

The present findings on the male dominance exhibited by *W. attu* are consistent with previous reports on the behavior of male dominance in the species *Clarias batrachus*, commonly known as the walking catfish [25]. However, these findings have now been further substantiated by numerous recent trials, which have demonstrated a male-dominated reproductive strategy in captivity [21,26,27]. It has been observed that male pursuit behavior is a common and persistent phenomenon during the courtship period. Furthermore, repeated chasing behaviors have been noted prior to spawning. A quantitative analysis was conducted, revealing that the behavior of chasing was repeated several times per trial and punctuated by brief behavioral events (2.05–5.15 s; Figure 4). This finding indicates that the behavior in question is characterized by a sequential and repeated pattern of interaction, as opposed to individual events. This finding suggests that the functional role of the male-driven stimulation that is sustained is involved in the synchronization of spawning (Table 5). The hypothesis that physical stimulation from the male is essential for aligning the female’s ovulation with the moment of fertilization is one that merits further investigation.

The hypothesis that the U-shaped wrapping and the persistent chasing behavior exhibited by *W. attu* serve a functional purpose in spawning, as opposed to serving a mere behavioral display, is substantiated by the available evidence. Equivalent stereotyped clasping behavior has also been described in Amur catfish (*Silurus asotus*), where adherence to the close body posture allows release of gametophytes to occur in synchrony and increases the efficiency of fertilization [28]. Analogous mechanical interactions in the spawning process have been observed in bighead catfish (*Clarias macrocephalus*), which also indicates that temporary but accurate physical interaction promotes external fertilization success [29]. The male dominance pattern is also evident in *Heteropneustes fossilis* [30]. The absence of parental care in *W. attu*, as well as the decreased hatching success when breeders remained in the tank, may be attributable to mechanical egg disturbance or opportunistic predation. This behavior has been reported frequently in a number of catfishes, including *C. batrachus* and the butter catfish (*Ompok bimaculatus*). Furthermore, the increase in hatching can be explained by adhesive egg ecology, in which better aeration and lower microbial colonization lead to embryo survival [20,27]. It can be concluded from these observations that the reproductive behavior exhibited by *W. attu* falls within the general trend observed among other species of silurid catfish. The former generally experience external fertilization and spawning devoid of egg guarding.

### 4.2. Species-Specific Courtship Behavior

In the case of *W. attu*, a 9–10 h latent period to courtship onset has been observed following the administration of hormones. This is within the extensive range documented in silurids; however, it does not correspond with that of certain related species. The Malabar barb (*Horabagrus nigricollaris*) has been observed to initiate courtship at an earlier stage, at 8–9 h [27]. The mating behavior of the long-whiskered catfish (*Mystus gulio*) has been observed to commence within a duration of five hours [31]. In contrast, the duration of *C. batrachus* is significantly longer, with an approximate range of 16 to 20 h [25]. These differences emphasize the necessity to devise hormonal regimens tailored to specific species for utilization in commercial hatcheries.

### 4.3. Absence of Parental Care

It has been observed that *W. attu* exhibits a lack of parental care. This is in contrast to other species of freshwater fish. The spotted snakehead (*Channa punctatus*) is an example of a species that exhibits strong male-guarded behavior and has a tendency to cluster its eggs [32]. In contrast, *O. bimaculatus* and stinging catfish (*Heteropneustes fossilis*) exhibit minimal or no evidence of egg care [20,30]. The present study has observed that the presence of brooders within the tank has been associated with a decline in the hatching rate. This phenomenon is associated with egg predation or mechanical disturbance, which is prevalent among cannibalistic catfishes such as *C. batrachus* [25]. It can be hypothesized that the increased fertilization rate for T1 was probably associated with improved synchronization of the spawning process and efficient release of gametes during the courtship period. Fertilization is an external process that occurs immediately after spawning. Consequently, this phenomenon is not related to the presence of brooders following egg release. Nevertheless, it has been hypothesized that the presence of brooders may result in increased egg disturbance or mechanical damage, which consequently leads to a decline in hatching success. *W. attu* is a non-guarding broadcast-type spawner due to an absence of nest construction, guarding, and larval protection.

### 4.4. Ecological and Evolutionary Environment of Male Bias

The high degree of male dominance evident in *W. attu* is hypothesized to be indicative of the fact that male competition and female fecundity dictate reproductive success in the species. This kind of male-biased behavior has been observed in bullseye catfish (*Horabagrus brachysoma*) [33]. In contrast, *H. nigricollaris* exhibits female courtship behavior [26]. The observed variation in species suggests that the roles assumed during the courtship process are contingent on ecological factors. These include the selection of spawning sites, the turbulence of the habitat, the risk of predation, and the organization of mating systems [34,35].

### 4.5. Plastic Strings Have Been Demonstrated to Be Efficient Substrates

The present study demonstrates the usefulness of plastic strings as adhesive substrates. The findings revealed that the fertility and hatching rates on plastic strings were significantly higher than those observed on aquatic vegetation. The hypothesis that this is the result of improved aeration, reduced microbial load, and a more homogeneous egg-binding position is one that merits further investigation. Catfish have been observed to demonstrate a clear preference for certain substrates. For instance, *H. nigricollaris* exhibits a preference for oviposition in the lower region of the spawning mops [26]. In contrast, *H. fossilis* and *O. bimaculatus* exhibit minimal substrate-type dependence [20,30]. In the case of *W. attu*, the utilization of synthetic substrates has been demonstrated to replicate natural surfaces, thereby enhancing the efficiency of the hatchery. This observation suggests that the utilization of plastic strings constitutes a pragmatic and efficacious substitute for natural vegetation in the context of *W. attu* spawning in captivity.

### 4.6. Seasonal Spawning Patterns and Reproductive Ethology of W. attu

The reproductive parameters observed in the present investigations suggest that *W. attu* has mostly a seasonal and single annual spawning strategy (Table 14). As reported in previous population studies of this species, peak spawning activity occurred from June to August. Shendge [36] recorded a single spawning season, which occurred from May to November. In contrast, Ghanbahadur [37] documented a single spawning season, which took place in June and July. The comparatively brief period of peak spawning activity documented in the present study may be attributable to geographic location, environmental factors, or the time and length of sampling duration across various studies. The spawning temperature obtained in the present study (27.53 ± 0.33 to 29.50 ± 0.58 °C) was also similar to that reported by Raizada [38] in captive breeding and early developmental studies. The findings indicate a distinct seasonal pattern in *Wallago attu* reproduction, with spawning activity concentrated during the warmer months.

Research into the ethology and induced spawning of *W. attu* remains limited due to the species’ vulnerable status and restricted broodstock availability. Consequently, the formation of comparative behavioral information within the family Siluridae is still ongoing. Consequently, the present study is regarded as a fundamental document on courtship sequence and captive reproductive systems, which can serve as a benchmark for subsequent ecological research and hatcheries.

### 4.7. Comparison of Fertilization, Hatching, and Hormonal Effects with Other Catfishes

The rates of fertilization and hatching observed in the present study (70.77–88.12% and 67.78–89.28%, respectively) were comparable with or better than those of other catfishes obtained with the same category of hormones. In the study of *Clarias gariepinus*, no significant difference in fertilization and hatching success was observed following treatment with Ovaprim [5,6]. The study revealed a latency period of 9–10 h, which is analogous to that observed in *H. nigricollaris* (9–10 h) [27] and *M. gulio* (5 h) [31], and significantly shorter than that reported for *C. batrachus* (16–20 h) [25]. With regard to the application of hormones, the following dosages were determined to be both effective and convenient: 0.5 mL kg^−1^ for females and 0.2 mL kg^−1^ for males. Moreover, the elimination of the need to kill males as part of other catfish breeding techniques based on testicular sperm was also a consequence of this method [1,3,6]. It has been demonstrated that alternative GnRH protocols may be more cost-effective [1,5]. However, Ovaprim is a ready-to-use solution that has been shown to deliver consistent results. This is of particular importance for species such as *W. attu*, which are considered vulnerable due to their limited number of broodstock. In summary, the present protocol has been demonstrated to provide reproductive performance that is comparable to that observed in other catfish breeding systems. Furthermore, it has been shown to address the issue of the loss of male brooders in captive breeding programs.

## 5. Conclusions

This is the first detailed behavioral description of courtship and spawning in *W. attu* under captive conditions worldwide. Although induced spawning of *W. attu* has been documented in other regions, no previous study has recorded the courtship sequence, male-driven chasing, U-shaped body wrapping, and simultaneous release of gametes as described here. In the absence of any parental care exhibited by *W. attu*, the removal of the brooders post-spawning resulted in a significant enhancement in hatching success. The findings of the study demonstrated that the utilization of plastic strings as spawning substrates resulted in enhanced fertilization and hatching rates when compared with aquatic weeds. This knowledge is of considerable practical and costeffective value and will serve as a baseline for the optimization of induced breeding protocols and hatchery management of this vulnerable fish. It is evident that the provision of a viable strategy for reducing the impact of wild populations and promoting sustainable aquaculture development is of significant benefit to hatchery managers and conservationists.

## Figures and Tables

**Table 1:** Ingredient composition and crude protein contribution of the commercial diet fed to *W. attu* broodstock.

Ingredient	Inclusion (kg)	Crude Protein (%)	Contribution of Ingredient in CP
Fish meal	34	60	20.4
Poultry by product meal	18	60	10.8
Soybean meal	26	46	11.96
Corn gluten meal	10	60	6
Wheat flour	3	12	0.36
Rice polish/bran	2.5	14	0.35
Fish oil	3	0	0
Dicalcium phosphate (DCP)	1	0	0
Vitamin–mineral premix	1	0	0
CMC/binder	0.5	0	0
Salt	0.5	0	0
DL-Methionine	0.3	0	0
L-Lysine	0.2	0	0
Total	100		49.87

**Table 2:** Details of the experimental setup for *W. attu* spawning under captivity conditions.

Set.	Description	T1	T2	P0	P1
1	Tank shape	Circular	Circular	Circular	Circular
2	Tank type	Cemented	Cemented	Cemented	Cemented
3	Mesh type	PVC + Nylon Mesh	PVC + Nylon Mesh	PVC + Nylon Mesh	PVC + Nylon Mesh
4	Tank height	4 feet	4.0 feet	4 feet	4 feet
5	Water inlet diameter (bottom)	0.75 inch	0.75 inch	0.75 inch	0.75 inch
6	Water inlet diameter (surface)	0.75 inch	0.75 inch	0.75 inch	0.75 inch
7	Volume of water	1100–1200 L	1100–1200 L	1100–1200 L	1100–1200 L
8	Outlet size (diameter)	2.5 inch	2.5 inch	2.5 inch	2.5 inch

**Table 3:** Fish weight and dose administration in male and female fish for *W. attu* spawning under captivity conditions.

Treatment	Sex	Fish Weight (kg)	Hormonal Dose (mL)
T1		3.30 ± 0.28 ^a^	1.44 ± 0.40 ^a^
T2		2.82 ± 0.25 ^b^	1.24 ± 0.35 ^b^
*p*-value		0.0012	0.0021
	Male	2.48 ± 0.11 ^b^	0.50 ± 0.02 ^b^
	Female	3.63 ± 0.14 ^a^	2.18 ± 0.08 ^a^
*p*-value		<0.0001	<0.0001
T1	Male	2.70 ± 0.10 ^c^	0.54 ± 0.02 ^c^
	Female	3.90 ± 0.12 ^a^	2.34 ± 0.07 ^a^
T2	Male	2.27 ± 0.09 ^c^	0.45 ± 0.02 ^d^
	Female	3.37 ± 0.09 ^b^	2.02 ± 0.05 ^b^
*p*-value		<0.0001	0.0154

Means within a column with different superscript letters are significantly different (*p* ≤ 0.05). T1: presence of fish brooders inside the fish tank after egg release; T2: removing brooders from the fish tank after egg release.

**Table 4:** Total length (cm) of male and female *W. attu* broodstock used in the brooder presence treatments.

Sex	T1	T2	*p* Value
Male	77 ± 4.36	72 ± 3.61	0.21
Female	85.67 ± 1.53	79.33 ± 2.52	0.2

T1 = brooders retained after egg release; T2 = brooders removed after egg release. Values are presented as mean ± SD; *p*-values indicate differences between treatments within each sex.

**Table 5:** Age (months) of male and female *W. attu* broodstock used in the brooder presence treatments.

Sex	T1	T2	*p* Value
Male	25 ± 1	26.67 ± 0.58	0.067
Female	25.67 ± 0.58	23.67 ± 1.53	0.1

T1 = brooders retained after egg release; T2 = brooders removed after egg release. Values are presented as mean ± SD; *p*-values indicate differences between treatments within each sex.

**Table 6:** Body weight and hormone dose administered to male and female *W. attu* broodstock during induced spawning using aquatic weeds vs. plastic strings as spawning substrates.

Treatment	Sex	Fish Weight (kg)	Hormonal Dose (mL)
P0	male	2.90 ± 0.22	1.25 ± 0.34
P1	female	3.07 ± 0.22	1.31 ± 0.35
*p*-value		0.3599	0.4522
	male	2.55 ± 0.13 ^b^	0.51 ± 0.03 ^b^
	female	3.42 ± 0.09 ^a^	2.05 ± 0.06 ^a^
*p*-value		<0.0001	0.0010
P0	male	2.43 ± 0.15 ^b^	0.49 ± 0.03 ^b^
	female	3.37 ± 0.09 ^a^	2.02 ± 0.05 ^a^
P1	male	2.67 ± 0.23 ^b^	0.53 ± 0.05 ^b^
	female	3.47 ± 0.19 ^a^	2.08 ± 0.11 ^a^
*p*-value		<0.0001	0.0063

Means within a column with different superscript letters are significantly different (*p* ≤ 0.05). P0 = aquatic weeds as substrate for adhesive eggs; P1 = plastic strings as substrate for adhesive eggs.

**Table 7:** Total length (cm) of male and female *W. attu* broodstock used in the substrate treatments.

Sex	P0	P1	*p* Value
Male	72 ± 5.29	75.33 ± 3.79	0.425
Female	83 ± 2.65	85 ± 5.29	0.59

P0 = aquatic weeds; P1 = plastic strings. Values are presented as mean ± SD; *p*-values indicate differences between treatments within each sex.

**Table 8:** Age (months) of male and female *W. attu* broodstock used in the substrate treatments.

Sex	P0	P1	*p* Value
Male	24 ± 1	27 ± 2.65	0.14
female	29.33 ± 0.58	30.67 ± 1.528	0.23

P0 = aquatic weeds; P1 = plastic strings. Values are presented as mean ± SD; *p*-values indicate differences between treatments within each sex.

**Table 9:** Physicochemical parameters recorded during the research trial of *W. attu* spawning under captivity conditions. PDA = post-dose administration.

Time (Minutes)	Temperature (°C)	Nitrate (mg/L)	Dissolved Oxygen (Aeration) (mg/L)	Electrical Conductivity (µS/cm)
0 PDA	28.30 ± 0.21	1.76 ± 0.15	4.87 ± 0.35	2.03 ± 0.01
30 PDA	29.50 ± 0.58	1.57 ± 0.12	5.76 ± 0.32	2.02 ± 0.06
60 PDA	28.67 ± 0.88	1.74 ± 0.12	5.92 ± 0.39	2.06 ± 0.02
90 PDA	27.53 ± 0.33	1.69 ± 0.17	6.11 ± 0.47	2.12 ± 0.04
120 PDA	28.51 ± 1.03	1.58 ± 0.15	5.76 ± 0.32	2.07 ± 0.03
150 PDA	27.80 ± 0.61	1.60 ± 0.09	5.82 ± 0.42	2.04 ± 0.02
180 PDA	28.03 ± 0.29	1.62 ± 0.19	5.85 ± 0.31	1.95 ± 0.03
210 PDA	28.17 ± 0.88	1.54 ± 0.12	5.95 ± 0.30	2.09 ± 0.04
240 PDA	28.97 ± 1.45	1.38 ± 0.06	5.75 ± 0.32	2.16 ± 0.06
270 PDA	28.40 ± 0.49	1.62 ± 0.15	5.74 ± 0.32	1.98 ± 0.11
300 PDA	28.83 ± 1.20	1.48 ± 0.12	5.84 ± 0.31	2.07 ± 0.03
330 PDA	28.20 ± 1.14	1.58 ± 0.10	5.85 ± 0.30	2.09 ± 0.03
360 PDA	28.30 ± 1.01	1.48 ± 0.20	5.46 ± 0.39	2.03 ± 0.08
390 PDA	27.83 ± 0.88	1.78 ± 0.09	5.16 ± 0.33	2.05 ± 0.03
420 PDA	28.30 ± 1.08	1.54 ± 0.23	5.39 ± 0.30	2.02 ± 0.05
450 PDA	28.27 ± 0.88	1.53 ± 0.19	5.46 ± 0.18	2.05 ± 0.09
480 PDA	28.50 ± 0.58	1.75 ± 0.12	4.94 ± 0.33	2.09 ± 0.04
510 PDA	28.70 ± 0.61	1.75 ± 0.15	5.28 ± 0.29	1.98 ± 0.08
540 PDA	28.27 ± 1.16	1.47 ± 0.06	5.32 ± 0.13	1.98 ± 0.12
570 PDA	28.83 ± 0.88	1.71 ± 0.15	4.96 ± 0.30	2.12 ± 0.05
600 PDA	28.40 ± 1.25	1.65 ± 0.27	5.45 ± 0.17	1.95 ± 0.06
*p*-value	0.999	0.929	0.244	0.523

**Table 10:** Ethogram used for spawning and parental behavior of *W. attu* during induced breeding.

Behavior	Activity	Intensity	Description
Shy Behavior	X	High	Spawners never jumped and were found at the tank bottom, showing shy behavior at the start of the trial.
Pairing	X	High	Before the first courtship attempts, the male and female were lying motionless and separated on the bottom of the tank.
Coloration change			No color change was observed in male and female fish.
Swimming Pattern	X	Medium	The swimming pattern included side-to-side swimming and often in a circular motion.
Nest building			Spawners were sluggish, but no nest building was observed.
Aggressive behavior	X	Medium	Aggressive behavior was observed in the male. Jumping or fast activities were seen as the trial progressed.
Spawning Embrace	X	Low	The male fish was observed rubbing his body against the female fish’s abdomen.
Chasing	X	High	The male chased aggressively and forced the female fish to copulate.
Close contact	X	Medium	The male fish aligned himself with the female fish.
Biting Attempt			No biting attempt was observed during this study.
U-Shaped Bending	X	High	The male fish was observed bending his whole body around the female fish.
Circling	X	Low	Pairs were observed swimming in circles
Egg Release	X	Medium	Male fish positioned in a U shape till egg release by female fish. The released eggs were dispersed in the tank with tail flaps.
Guarding			No guarding was observed.

Behaviors expressed are marked with X.

**Table 11:** Courtship and mating ritual observations of *W. attu* during induced breeding.

Figure No.	Characteristics
Figure 1A,B	Male fish chasing female fish
Figure 1C–K	U-shaped bending of male fish around female fish
Figure 2A–H	Female fish started twisting her body and released eggs
Figure 3A–F	Male and female fish separated after egg release

**Table 12:** Comparison of reproductive potential for assessment of parental care of *W. attu* during induced breeding.

Treatment	Ovulation Time (Minutes)	Fertilization Rate (%)	Hatching Rate (%)
T1	648.33 ± 11.85	82.25 ± 2.44 ^a^	67.78 ± 1.78 ^b^
T2	643.33 ± 10.71	70.77 ± 2.50 ^b^	88.12 ± 0.26 ^a^
*p*-value	0.7699	0.0303	0.0003

Means within a column with different superscript letters are significantly different (*p* ≤ 0.05).

**Table 13:** Comparison of reproductive potential in treatments provided with aquatic weeds and plastic strings of *W. attu* during induced breeding.

Treatment	Ovulation Time (Minutes)	Fertilization Rate (%)	Hatching Rate (%)
P0	678.00 ± 10.41	75.16 ± 2.26 ^b^	69.53 ± 3.13 ^b^
P1	664.33 ± 12.68	86.42 ± 1.18 ^a^	89.28 ± 0.80 ^a^
*p*-value	0.4516	0.0115	0.0036

Means within a column with different superscript letters are significantly different (*p* ≤ 0.05).

**Table 14:** Reproductive parameters of *W. attu* recorded in the present study.

Reproductive Parameter	Present Study
Spawning pattern	Single
Spawning frequency	Once a year
Spawning period	June to August (maximum)
Spawning associated temperature	27.53 ± 0.33 to 29.50 ± 0.58 °C

## Data Availability

Data will be provided upon reasonable request from the authors.
